# Intra- and Inter-Individual Spectral Pattern Variability of sEMG in Elbow Flexor Motor Tasks

**DOI:** 10.3390/s26030878

**Published:** 2026-01-29

**Authors:** Piotr S. Wawryka, Ludwin Molina Arias, Grzegorz Frankowski, Patryk Ciężarek, Joanna Zyznawska, Jan T. Duda

**Affiliations:** 1 Department of Biocybernetics and Biomedical Engineering, Faculty of Electrical Engineering, Automatics, Computer Science and Biomedical Engineering, AGH University of Krakow, Mickiewicza 30, 30-059 Krakow, Poland; arias@agh.edu.pl; 2 Institute of Physiotherapy, Faculty of Health Sciences, Jagiellonian University Medical College, Badurskiego 19, 30-694 Krakow, Poland; grzegorz.frankowski@uj.edu.pl (G.F.); patryk.ciezarek@uj.edu.pl (P.C.); joanna.zyznawska@uj.edu.pl (J.Z.); 3 Motor Function Research Laboratory, Center for the Development of Therapies for Civilization and Age-Related Diseases, Jagiellonian University Medical College, Medyczna 7A, 30-688 Krakow, Poland; 4 Department of Automation and Robotics, University of Applied Science in Tarnow, Mickiewicza 8, 33-100 Tarnów, Poland; jdu@agh.edu.pl

**Keywords:** surface electromyography, spectral variability, elbow flexors, motor task, forearm orientation, distance-based metrics

## Abstract

Understanding intra- and inter-individual variability in muscle activation is essential for applications in rehabilitation, ergonomics, and motor control research. Surface electromyography (sEMG) provides a non-invasive tool to study these patterns by capturing the electrical activity of muscles. This study investigated the spectral pattern variability of sEMG signals recorded from the biceps brachii and brachioradialis during repeated near-maximal isometric elbow flexion tasks with supinated and neutral forearm postures. sEMG signals from 33 healthy adults were analyzed in the frequency domain to obtain power spectra for each repetition. Intra-individual variability was quantified by comparing each repetition to a participant-specific reference spectrum, while inter-individual variability was assessed by comparing these reference spectra across participants using distance-based metrics. Statistical analyses revealed systematic posture-dependent differences, with the neutral forearm posture generally exhibiting greater spectral variability than the supinated posture, particularly in the biceps brachii. These findings highlight potential posture-related trends in neuromuscular activation; however, they should be interpreted with caution, as variability may also reflect differences in contraction intensity, fatigue, or task-specific biomechanics.

## 1. Introduction

Surface electromyography (sEMG) has emerged as a powerful tool for investigating human neuromuscular function. By capturing the electrical signals generated by muscle fibers, sEMG provides insights into muscle activation patterns during voluntary movements, allowing for the analysis of both temporal and spectral features of muscle contractions [1]. Its ability to record electrical activity from broad muscle regions has made it valuable in clinical assessment, rehabilitation, performance monitoring, and human–machine interaction [2,3,4].

The elbow flexor muscles, particularly the biceps brachii (BB) and brachioradialis (BR), are essential for upper-limb function, contributing to tasks ranging from fine motor control to load lifting. Variations in forearm orientation have been shown to modify both the magnitude and distribution of muscle activation, reflecting the biomechanical and neuromuscular demands associated with each posture [5]. The effects of these postural changes on sEMG signals have been examined primarily through time-domain analyses, especially in studies of activation behavior and gesture-decoding performance [6]. For instance, Qing et al. [7] demonstrated that changes in forearm angle significantly affect classification accuracy, while Rumman et al. [8] introduced a dataset that explicitly incorporates multiple forearm orientations, highlighting the importance of accounting for posture-dependent variability when interpreting sEMG-based results.

Beyond time domain descriptors, spectral analysis offers an alternative representation of the signal by characterizing how sEMG energy is distributed across frequencies [9]. The Frequency-based perspective provides important insights into muscle function, enabling the quantification of physiological and biomechanical factors such as muscle fatigue, contraction intensity, and the neuromuscular conditions associated with changes in muscle–tendon geometry and motor unit recruitment strategies [10,11,12]. The value of spectral analysis has been demonstrated across a wide range of upper-limb neuromuscular tasks. For example, Costa-García et al. [13] showed that sustained isometric contractions induce a progressive shift of spectral energy from higher to lower frequencies as fatigue develops. Spectral decomposition methods, including principal component analysis and non-negative matrix factorization, have further revealed that the sEMG spectrum can often be represented by a small set of dominant components corresponding broadly to fast and slow motor unit action potentials [14]. Traditional frequency-domain metrics, such as mean power frequency (MNF) and amplitude-based indices (e.g., RMS), support their utility in quantifying fatigue and activity levels [15], while time–frequency approaches like the Short-Time Fourier Transform enable the characterization of spectral evolution during dynamic tasks, offering a more comprehensive depiction of muscle behavior under changing mechanical demands [16].

Methodological advances, including wavelet-based approaches, distance-based metrics, dimensionality-reduction techniques, and machine-learning methods, have substantially enriched the analysis of sEMG signals [17,18,19]. These developments enable a more detailed characterization of nonstationary signal components, multiscale spectral features, and complex interdependencies across muscles and tasks, thereby supporting investigations under a wide range of neuromuscular conditions, from healthy motor control to pathological states [20]. In parallel, the growing availability of publicly accessible sEMG repositories has provided valuable resources for improving reproducibility, benchmarking analytical pipelines, and facilitating cross-study comparisons, ultimately strengthening the methodological rigor and translational potential of sEMG-based research [21].

Despite extensive evidence that upper limb posture alters muscle activation and, consequently, the electrophysiological determinants that shape sEMG signals [22,23,24], the resulting changes in frequency-domain characteristics remain insufficiently characterized. In particular, it is still unclear how different upper limb postures or task types modulate spectral patterns within individuals, or how reproducible these posture-specific frequency features are across participants. Addressing these open questions requires evaluating spectral variability at both the intra-individual and inter-individual levels, as these complementary perspectives are essential to determine whether frequency-domain descriptors can function as reliable and generalizable neuromuscular markers [25,26,27].

In response to this gap, the present study investigates posture-dependent spectral variability in sEMG signals recorded from the BB and BR during two isometric elbow-flexion tasks performed under distinct forearm postures. We propose a unified analytical framework in which normalized average power spectra are computed for each muscle–task condition, and Minkowski-based distance metrics are employed to quantify both intra- and inter-individual consistency of spectral patterns. By jointly characterizing within-subject modulation and between-subject reproducibility, this approach advances beyond descriptive spectral analysis and provides a structured basis for identifying posture-specific sEMG frequency signatures. The resulting reference profiles contribute novel insights into how forearm orientation shapes sEMG spectral characteristics and offer practical value for future applications in neuromuscular assessment, ergonomics, and rehabilitation.

## 2. Methods

### 2.1. Subjects

Thirty-three healthy adults with no known history of neuromuscular or musculoskeletal disorders were recruited to participate in the study. All participants were informed about the experiment, and their participation was voluntary. Written informed consent was obtained prior to inclusion, and the participants retained the right to withdraw at any time without providing a reason. The study protocol was reviewed and approved by the Ethics Committee for Scientific Research of the Jagiellonian University Medical College in Krakow (decision no. 118.6120.75.2023, issued on 22 September 2023).

Participant demographic data are presented in Table 1.

The distribution of anthropometric characteristics across male and female participants was consistent with typical values for young adults, ensuring balanced representation in subsequent analyzes.

### 2.2. Data Acquisition Protocol

Each participant performed two motor tasks designed to activate the primary muscles involved in elbow flexion. Both tasks were executed sequentially within a single session. In the first task, participants pressed their open palms against the underside of a table, simulating an upward lifting motion without displacement, with the forearm fully supinated and the hand extended (Supinated open-hand task, SP). In the second task, the same pressing motion was performed with the hand clenched into a fist, maintaining firm contact with the table during the isometric effort, with the forearm in a neutral orientation (Neutral grip task, NT), allowing slight wrist flexion. In terms of the SFTR recording method convention (S—sagittal, F—frontal, T—transverse, R—rotation), the rotation of the forearm, affecting the radiocarpal joint, corresponded to approximately $0^{\circ }$ during SP and about $90^{\circ }$ during NT, while the elbow joint remained flexed at $90^{\circ }$ [28].

**Figure 1:**
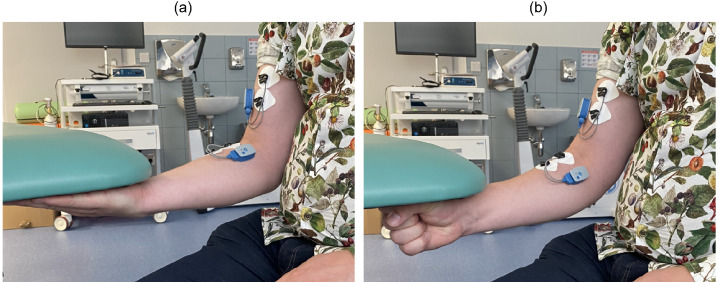
Motor tasks performed by the participants. (**a**) SP and (**b**) NT.

Each motor task consisted of 10 repetitions, with repetitions at one forearm orientation completed before moving to the other. Participants were instructed to exert maximal voluntary contraction (MVC) force during each repetition, maintaining the pressing effort for approximately 5 s, with short rest intervals between trials to allow for voluntary muscle relaxation. Figure 1 shows the motor tasks performed by the participants.

sEMG signals were recorded simultaneously from the biceps brachii (BB) and brachioradialis (BR) muscles of the dominant arm. These muscles were selected because of their primary role in elbow flexion during isometric contraction. Signal acquisition was performed using a Noraxon Ultium wireless EMG system (Noraxon Inc., Scottsdale, AZ, USA), which supports the simultaneous recording of multiple muscle groups.

Disposable pre-gelled Ag/AgCl surface electrodes (Skintact Premier F-301, Leonhard Lang GmbH, Innsbruck, Austria 30 mm diameter) were used for all recordings. Electrode placement followed the standardized recommendations of the International Society of Electrophysiology and Kinesiology (ISEK) as defined in the SENIAM guidelines [29]. To ensure consistency and reproducibility across participants, all electrodes were placed by the same trained investigator using a predefined anatomical landmark-based protocol, thereby ensuring strict adherence to ISEK/SENIAM standards.

For BB, electrodes were positioned along the line connecting the acromion to the cubital fossa, at one-third of the distance from the cubital fossa, with the electrode pair aligned parallel to the muscle-fiber orientation. For BR, placement followed the line connecting the lateral epicondyle of the humerus to the radial styloid process, at one-third of the proximal distance, over the thickest portion of the muscle. In both muscles, electrodes were placed with their edges touching, yielding a consistent center-to-center distance of 30 mm.

Before the experimental protocol, each participant completed a familiarization session to become accustomed to the testing setup and to ensure the correct execution of the isometric contractions.

Following data collection, a signal quality check was performed, and all recordings met the predefined criteria. Baseline noise levels remained consistently below 5 μV in a 5-s window, ensuring artifact-free recordings suitable for analysis in healthy individuals. All sEMG signals were sampled at 2 kHz using a 24-bit ADC with a dynamic resolution of 0.3 μV for signals ranging from 0 to 5.000 μV and 1.1 μV for signals ranging from 5.001 to 24.000 μV.

### 2.3. Processingof sEMG Signals

**Figure 2:**
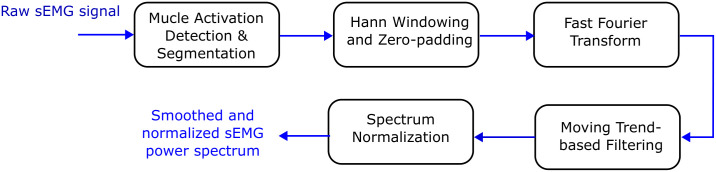
sEMG signal processing workflow applied to each raw recording.

The numerical processing of the acquired sEMG signals was performed in MATLAB R2023a (MathWorks, Natick, MA, USA) following a standardized analysis workflow. As shown in Figure 2, each raw sEMG recording was processed individually, and muscle isometric contractions (repetitions) were segmented and analyzed separately. Each segment was transformed into the frequency domain, smoothed, and used to obtain spectral estimates for each repetition.

#### 2.3.1. sEMG Segmentation

**Figure 3:**
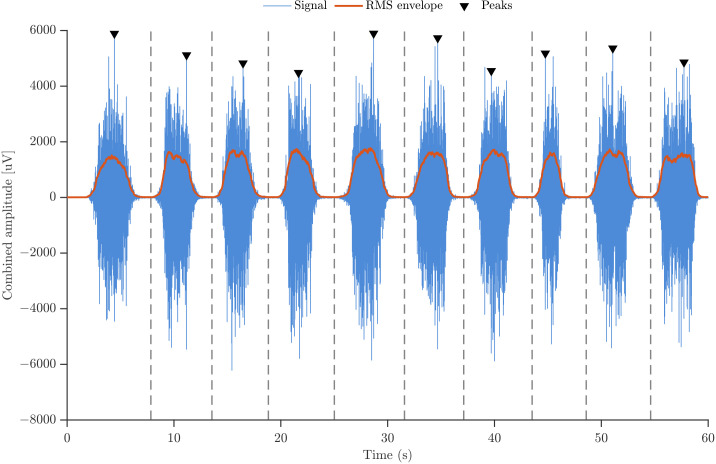
Illustration of the sEMG segmentation procedure.

The segmentation of sEMG signals was performed using a deterministic procedure that combines peak detection and envelope-based refinement to isolate individual contractions within each motor-task sequence. As illustrated in Figure 3, a reference trace was first constructed by summing the two EMG channels, assuming approximate phase synchrony and thereby enhancing the amplitude contrast during contractions. Candidate peaks corresponding to muscle actions were then identified using three constraints: (i) a minimum prominence of 500 μV to ensure that only well-defined contractions were detected; (ii) a minimum inter-peak distance of 0.35 s, consistent with the experimental pacing; and (iii) an amplitude threshold that enforced a minimum difference between each peak and its immediate neighbors, preventing minor oscillations or baseline fluctuations from being misclassified as contractions. These constraints ensured that only physiologically meaningful muscle actions were retained.

Once the candidate peaks were detected, the boundaries of each repetition were refined by computing the root-mean-square (RMS) envelope within the interval between consecutive peaks. The local minimum of this envelope was selected as the segmentation boundary, as it corresponds to a phase of minimal muscular activity between adjacent contractions. These envelope-derived boundaries, indicated by vertical dashed lines in Figure 3, provide a robust and physiologically meaningful criterion that minimizes overlap between neighboring contractions and ensures that each extracted segment contains a single muscle action.

#### 2.3.2. Windowing and Zero-Padding

After segmentation, each sEMG repetition was preprocessed prior to spectral analysis. Although the wireless sEMG system incorporates hardware-based measures to reduce power-line interference, a digital 50 Hz notch filter with a quality factor of 35 was applied to further attenuate residual mains contamination. This additional filtering step ensured that the subsequent spectral estimates predominantly reflected physiological sEMG activity, minimizing the influence of narrowband interference at the power-line frequency.

Following the segmentation stage, the resulting muscle-action segments were processed to ensure temporal consistency across repetitions. Because the duration of individual contractions varied slightly, the longest segment within each recording was first identified and used as the reference length. All other segments were then time-normalized by zero-padding until they matched this reference duration. This procedure preserved the intrinsic temporal structure of each contraction while enabling sample-by-sample comparability in subsequent analyzes. Moreover, zero-padding increased the frequency resolution of the spectrum without modifying the amplitude or phase characteristics of the original signal.

To further improve the quality of the spectral estimates, a Hann window was applied to each zero-padded segment prior to the frequency-domain transformation. Windowing attenuates discontinuities at segment boundaries, thereby reducing spectral leakage and suppressing side lobes in the resulting power spectrum. The combined use of zero-padding and Hann windowing ensured consistent, high-resolution, and leakage-minimized spectral representations across repetitions, muscles, and motor tasks.

#### 2.3.3. Fast Fourier Transform

Each preprocessed segment was converted into the frequency domain using the fast Fourier transform (FFT). For a discrete-time signal x[n] of length *N*, the transform is given by:

(1) $$X\left[m\right]=\sum_{n=0}^{N-1}x\left[n\right]\;e^{-j2\pi mn/N}$$

where m=0,…,N−1.

The corresponding power spectrum was computed as follows:

(2) $$P\left[f_{m}\right]={\left|X\left[m\right]\right|}^{2}$$

where $f_{m}=mf_{s}/N$ denotes the frequency associated with the *m*-th bin.

Using a uniform segment length ensured an identical frequency grid across repetitions and conditions, enabling direct comparison of the resulting power spectra.

#### 2.3.4. Moving Trend-Based Filtering

To obtain a noise-robust representation of the sEMG power spectra, smoothed power spectra were computed using a Moving Trend Filter (MTF) [30]. The MTF estimates, at each frequency bin $f_{m}$, a local low-order polynomial trend of the discrete spectrum S[m] within a sliding window, thereby attenuating fine-scale spectral fluctuations while preserving the overall spectral shape relevant for sEMG analysis.

Formally, for a window centered at $m_{k}$, the polynomial coefficients ${\left\{a_{r}\right\}}_{r=0}^{p}$ are obtained by solving the local least-squares problem:

(3) $${\left\{a_{r}\right\}}_{r=0}^{p}=\arg\min_{\left\{a_{r}\right\}}\sum_{k=1}^{K}{\left(S\left[m_{k}\right]-\sum_{r=0}^{p}a_{r}\;u_{k}^{r}\right)}^{2}$$

where *K* is the number of samples within the local window used for the polynomial approximation, $m_{k}$ is the corresponding frequency-bin index, $u_{k}$ is the normalized window coordinate for index $m_{k}$, and *p* is the polynomial order.

The smoothed signal value at point *n* is obtained by averaging the *K* values of the polynomial approximation over a sliding window from n−K+1 to n+K, corresponding to a total of M=2K+1 samples. In this study, a third-order polynomial (p=3) was employed. This procedure yields an amplitude characteristic with favorable low-pass properties, with its first minimum (close to zero) located at point $f_{Amin}=\frac{2}{K\;dt}$.

The smoothing window spanned 180 frequency bins, corresponding to 180,Δf, where $\Delta f=f_{s}/N$. The filter parameters were selected according to established recommendations [31], ensuring effective suppression of spectral noise while maintaining the essential spectral features of the sEMG signal.

#### 2.3.5. Spectrum Normalization

Normalization was applied exclusively in the frequency domain to ensure consistent comparisons across repetitions, muscles, and motor tasks, emphasizing relative spectral characteristics rather than absolute amplitude. Time-domain amplitude normalization, including MVC-based scaling, was intentionally omitted to avoid introducing variability unrelated to the intrinsic spectral content.

Two normalization strategies were employed:

- Maximum power Normalization: Each power spectrum was scaled by its peak value within the analyzed frequency range, ensuring that the dominant frequency component equals unity and facilitating shape-based comparisons.
  (4) $$P\left[f_{m}\right]=\frac{P[f_{m}]}{\max_{m}P\left[f_{m}\right]}$$
  where $P[f_{m}]$ is the normalized power spectrum.
- Total Energy Normalization: The power spectrum was normalized so that its discrete energy equals one, yielding a probability-like representation of how the spectral content is distributed across frequencies:
  (5) $$P\left[f_{m}\right]=\frac{P[f_{m}]}{\sum_{m=0}^{N-1}P\left[f_{m}\right]}$$
  where the denominator represents the total energy of the smoothed spectrum across all frequency bins.

### 2.4. Variability Analysis

Variability was quantified in the spectral domain using the normalized power spectra obtained from each EMG activation. Since two muscles (BB and BR) were recorded under two forearm postures (SP and NT), each participant contributed four sets of power spectra corresponding to the conditions BB/SP, BB/NT, BR/SP, and BR/NT. Each set contained ten repetitions of the same isometric task, acquired under identical experimental conditions.

Because repeated contractions within a condition reflect the same intended motor execution, the 10 normalized power spectra for each participant and condition were first averaged to obtain a subject-specific reference spectrum:

(6) $${\bar{P}}_{s,c}\left[f_{m}\right]=\frac{1}{10}\sum_{i=1}^{10}{\tilde{P}}_{s,c}^{\left(i\right)}\left[f_{m}\right]$$

where *s* indexes the participant, *c* the muscle/posture condition, and ${\tilde{P}}_{s,c}^{\left(i\right)}\left[f_{m}\right]$ the normalized power spectrum of the *i*-th repetition. These reference spectra constitute compact descriptors of each participant’s characteristic spectral pattern for each task.

#### 2.4.1. Intra-Subject Variability

Intra-subject variability was quantified by measuring how much each repetition deviated from the corresponding subject-specific reference spectrum. For each participant *s* and condition *c*, the deviation of the *i*-th repetition was computed using Minkowski distances, defined as:

(7) $$D\left(x,y\right)={\left(\sum_{k=1}^{N}{\left|x_{k}-y_{k}\right|}^{\;l}\right)}^{1/l}$$

where *l* determines the order of the norm.

Three commonly used cases were considered: l=1 (Manhattan distance, $L^{1}$), l=2 (Euclidean distance, $L^{2}$), and l→∞ (Chebyshev distance, $L^{\infty }$). For the general Minkowski metric of order *l*, the intra-subject distance for the *i*-th repetition was computed as:

(8) $$d_{s,c}^{\left(i\right)}={\left(\sum_{k=1}^{N}{\left|{\tilde{P}}_{s,c}^{\left(i\right)}\left[f_{m}\right]-{\bar{P}}_{s,c}\left[f_{m}\right]\right|}^{l}\right)}^{1/l}$$

This operation resulted in 10 distances per participant and condition. These values were summarized using their means, standard deviations, and coefficients of variation, providing a compact characterization of how consistently each participant reproduced the same power spectrum across repeated contractions.

To assess whether forearm posture affects intra-subject variability, distances obtained under the SP and NT postures were compared for each muscle using the Wilcoxon signed-rank test. The null hypothesis ($H_{0}$) assumed no difference between postures. Statistical significance was determined based on the resulting *p*-value, indicating whether forearm posture had a significant effect on spectral reproducibility.

#### 2.4.2. Inter-Subject Variability

Inter-subject variability was quantified by computing pairwise spectral distances between participants performing the same muscle–posture condition. For each condition *c* and normalization scheme, each participant contributed a reference spectrum ${\bar{P}}_{s,c}\left[f_{m}\right]$, obtained by averaging the 10 repetitions of that condition. Pairwise dissimilarity was then computed using the Minkowski distance over the common frequency grid:

(9) $$d_{c}\left(s_{1},s_{2}\right)={\left(\sum_{k=1}^{N}{\left|{\bar{P}}_{s_{1},c}\left[f_{m}\right]-{\bar{P}}_{s_{2},c}\left[f_{m}\right]\right|}^{l}\right)}^{1/l}$$

where $s_{1}$ and $s_{2}$ denote the participants.

To evaluate whether forearm posture influenced inter-subject spectral variability, inter-subject distances obtained under the SP and NT postures were compared for each muscle using paired observations. The null hypothesis ($H_{0}$) assumed no systematic difference in inter-subject spectral distances between postures. Statistical significance was assessed based on the resulting *p*-value, indicating whether forearm posture significantly affected inter-subject spectral dissimilarity.

Beyond hypothesis testing, the full distributions of inter-subject distances were examined using probabilistic modeling. Histograms were fitted with Gaussian, Laplace (double exponential), and Maxwell–Boltzmann distributions, with parameters estimated via goodness-of-fit assessed using the chi-square test. This approach characterizes not only the central tendency of inter-subject variability but also the shape and dispersion of its underlying statistical structure.

## 3. Results

The results of the analysis conducted are presented below. For the purpose of clarity, only the results obtained using $L^{2}$ are shown graphically. The reader is referred to the Supplementary Materials for results obtained with other distance metrics.

### 3.1. Processing of sEMG Signals

**Figure 4:**
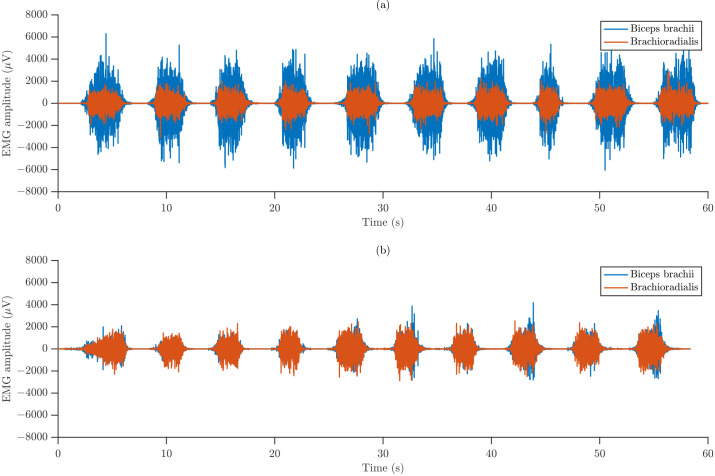
Representative waveforms of raw sEMG signals recorded from BB and BR during (**a**) SP and (**b**) NT.

Figure 4 illustrates representative raw sEMG recordings obtained from a single participant, showing the isometric contractions performed under the two forearm orientations for both monitored muscles. The recordings reveal differences in amplitude between the muscles and postures, indicating possible posture-dependent recruitment strategies and distinct levels of neuromuscular activation.

**Figure 5:**
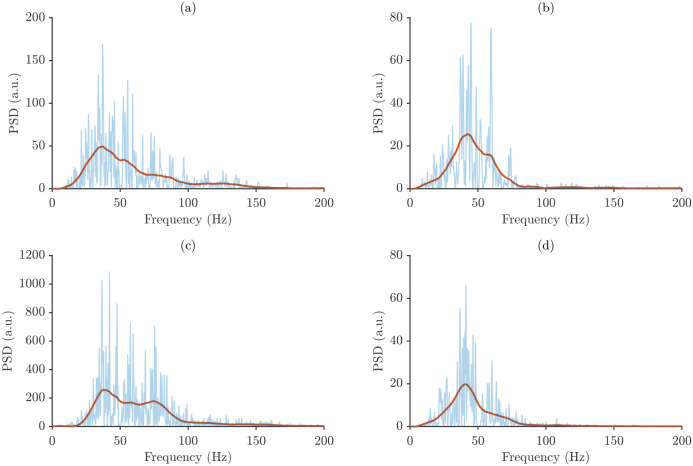
Representative power spectra from a single repetition of one participant across the four muscle–posture combinations. Subfigure (**a**) BB/SP, (**b**) BR/SP, (**c**) BB/NT, and (**d**) BR/NT.

After segmenting the sEMG recordings, the signal fragments corresponding to individual muscle activations were extracted and transformed into the frequency domain. For each segment, the power spectrum was computed and subsequently smoothed using the Moving Trend Filter (MTF). Figure 5 shows representative power spectra for each muscle and forearm posture from a single participant.

Figure 5 reveals distinct spectral profiles for each muscle and forearm posture. Although power spectra recorded from the same muscle tend to exhibit broadly similar shapes, clear differences emerge between postures, underscoring the sensitivity of the spectral content to changes in forearm orientation. These posture-dependent spectral shifts form the basis for the subsequent intra- and inter-subject variability analyzes.

### 3.2. Variability Analysis

**Figure 6:**
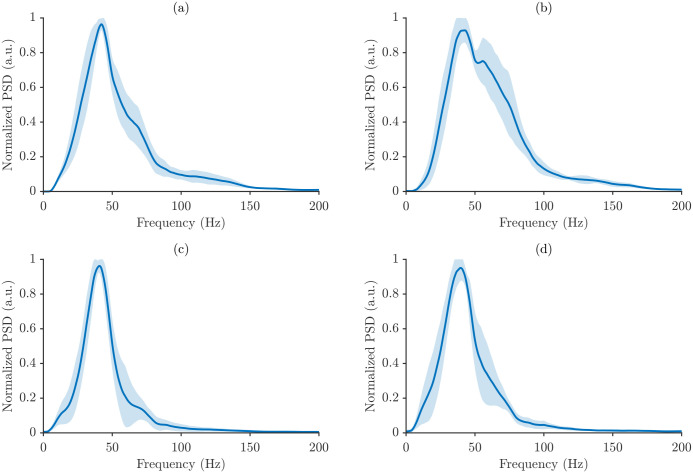
Average power spectra of a representative participant under maximum power normalization for all muscle–task conditions: (**a**) BB/SP, (**b**) BB/NT, (**c**) BR/SP, and (**d**) BR/NT. Shaded area represents the mean ± STD.

**Figure 7:**
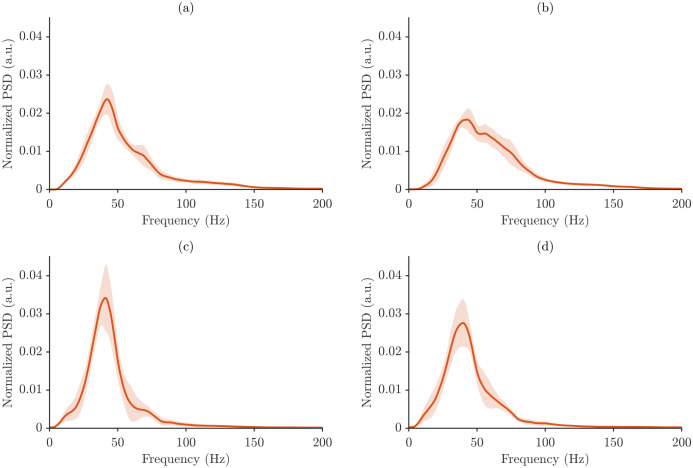
Average power spectra of a representative participant under total energy normalization for all muscle–task conditions: (**a**) BB/SP, (**b**) BB/NT, (**c**) BR/SP, and (**d**) BR/NT. Shaded area represents the mean ± STD.

Figure 6 presents the average power spectra of a representative participant for all muscle–task combinations under maximum power normalization. For each condition, the average power spectrum is shown together with a shaded band representing one standard deviation across repetitions, which allows for direct visualization of within-participant variability. Figure 7 shows the analogous average power spectra computed using total energy normalization.

Figure 6 and Figure 7 highlight differences in spectral signatures across motor tasks and muscles, with SP and NT subtly distinguished by changes in spectrum shape and dispersion. The normalized power spectral density (PSD) is shown for the four analyzed muscle/posture conditions, and in all cases, the spectra are dominated by low-frequency energy, followed by a gradual decline at higher frequencies, reflecting stable sensor performance and repeatable measurement conditions.

The employed normalization strategies strongly influenced the appearance of the spectra. Maximum power normalization produced more compact spectra with lower variability across repetitions, emphasizing dominant peaks while minimizing smaller fluctuations. Total energy normalization, in contrast, yielded broader spectra that were more sensitive to subtle variations in power across frequency bins.

The key difference between the spectra lies in their magnitudes. Mean energy spectra show lower normalized PSD values, representing the average distribution of signal energy, whereas maximum mean spectra show higher PSD amplitudes, highlighting the strongest energy contributions. Importantly, despite these differences in magnitude, the overall spectral shape and dominant frequency components remained consistent across all cases.

#### 3.2.1. Intra-Subject Spectral Variability

**Figure 8:**
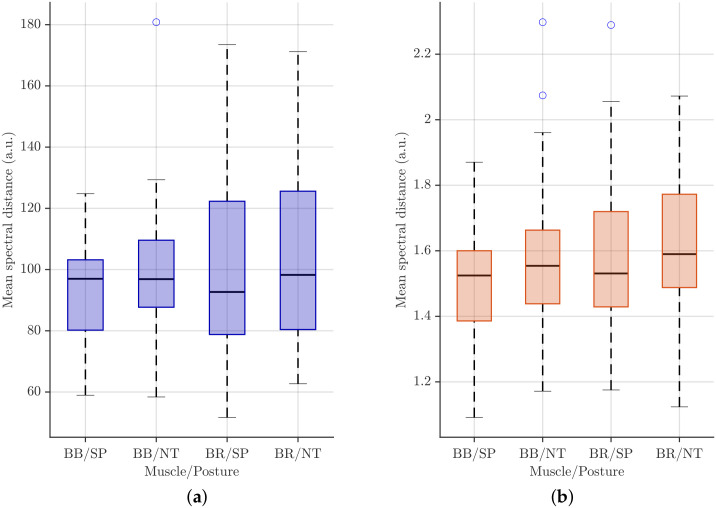
Boxplots showing intra-individual spectral variability across muscle–task categories. (**a**) Maximum power normalization and (**b**) Total energy normalization. Circles denote outliers.

Figure 8 shows the boxplots of intra-subject distances for all participants, separated by muscle and task. These boxplots illustrate the magnitude and dispersion of variability across repetitions, allowing for a visual comparison between the SP and NT conditions.

Across all muscle–task combinations and normalization schemes, the median spectral distance for NT is consistently higher than that for SP, indicating greater intra-individual variability. This effect is particularly pronounced in the BR, where longer boxes reflect larger interquartile ranges compared to those of the BB.

Normalization strongly influenced the distributions. Under maximum power normalization (Figure 8a), NT shows higher medians and wider IQR than SP, with the BR exhibiting the greatest spread, suggesting that peak-power scaling amplifies variability across repetitions. Total energy normalization (Figure 8b), in contrast, produces more compact distributions while preserving relative differences between postures and muscles, stabilizing intra-individual spectral features.

Quantitative descriptors, such as the mean, standard deviation (STD), Coefficient of variation (CV), minimum, and maximum for each muscle–task condition and each distance metric, are reported in Table 2 and Table 3.

The quantitative descriptors reported in Table 2 and Table 3 provide a detailed overview of intra-individual spectral variability across muscle–task conditions and distance metrics under maximum power and total energy normalization, respectively. Across all cases, the median spectral distance for NT was generally higher than that for SP, reflecting greater variability. This effect was particularly pronounced in the BR, which also exhibited longer interquartile ranges than the BB, indicating broader dispersion. Maximum power normalization amplified these differences, yielding higher means, STD, and coefficients of variation (CV), whereas total energy normalization produced more compact distributions with reduced dispersion.

Given the paired design of the experiment and assuming the absence of normality in the distributions of spectral distances, statistical comparisons between SP and NT were performed using the Wilcoxon signed-rank test. The resulting *p*-values for each metric and muscle pair are listed at the bottom of Table 2 and Table 3. During the test, a significance threshold of α=0.05 was adopted.

Statistical comparisons using the Wilcoxon signed-rank test confirmed that posture-dependent differences were significant under maximum power normalization for several muscle–distance combinations. Specifically, $L^{2}$ distances revealed significant effects for both BB (p=0.0296) and BR (p=0.0168); $L^{1}$ distances were significant for BR only (p=0.0197), and $L^{\infty }$ distances reached significance for BR (p=0.0073), while BB showed a marginal trend (p=0.0573). In contrast, total energy normalization yielded no statistically significant differences for any muscle or distance metric (p≥0.0778), indicating that this normalization stabilizes intra-subject distances and reduces posture-related effects.

#### 3.2.2. Inter-Subject Spectral Variability

Inter-subject variability was quantified by computing pairwise spectral distances between participants. For each muscle–task category, the average spectrum of every participant was compared with those of all other participants, producing a distribution of between-subject distances that reflects how heterogeneous the spectral patterns are across the cohort.

**Figure 9:**
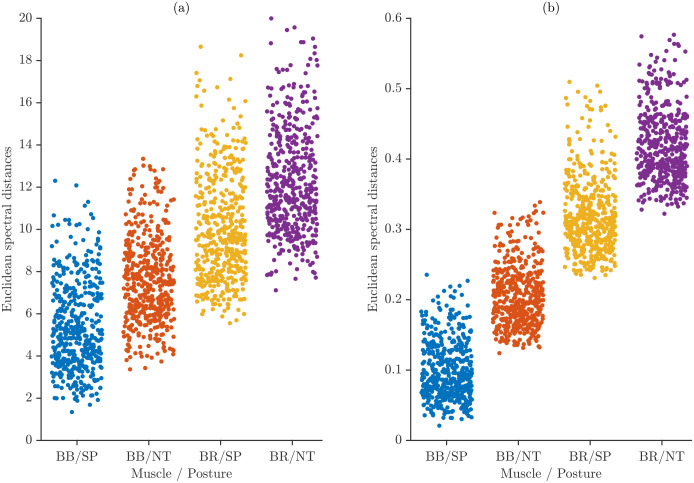
Pairwise inter-subject spectral distances for all muscle–task categories: (**a**) maximum power normalization and (**b**) total energy normalization. Vertical offsets are applied only for visual separation of categories.

Figure 9 shows these pairwise distances for all muscle–task categories, displayed separately for maximum power normalization and total energy normalization. Each point represents the distance between the spectra of two distinct participants belonging to the same category. To facilitate visual separation of the four muscle–task groups, the corresponding point clusters were vertically offset using the distances between the category-level average spectra. These offsets do not alter the pairwise distance values themselves; they simply ensure that the point clouds do not overlap.

The vertical spread within each group reflects the true inter-subject variability for that category, whereas the spacing between groups results only from the applied visual offsets and does not represent actual spectral separation between categories. The distributions show subtle differences in inter-subject variability across muscle–task conditions and normalization schemes.

**Figure 10:**
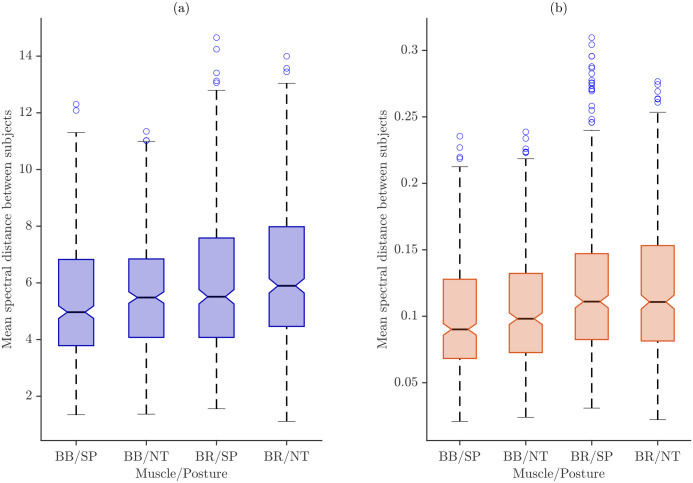
Boxplots showing inter-individual spectral variability across muscle–task categories. (**a**) Mximum-power normalization and (**b**) Total energy normalization. Circles denote outliers.

Figure 10 illustrates the inter-individual spectral variability across muscle–task categories using boxplots for the two different normalization strategies.

Boxplots for maximum power normalization (Figure 10a) show that median spectral distances increase from BB/SP to BR/NT, indicating higher inter-subject variability for BR compared with BB and for the NT posture relative to SP. The IQRs are relatively wide, particularly for BR, reflecting substantial between-subject variability, and several high-value outliers suggest that a subset of participants exhibits markedly different spectral characteristics under this normalization.

In contrast, total energy normalization (Figure 10b) produces lower overall distance values and more compact distributions. Medians across muscle–posture categories are closer, and IQRs are narrower than those under maximum power normalization, indicating reduced inter-individual variability. While BR still shows slightly higher medians and variability than BB, the differences between SP and NT postures are less pronounced, and outliers are fewer and less extreme, highlighting the stabilizing effect of total energy normalization on spectral distance measures.

Table 4 and Table 5 report descriptive statistics, providing a quantitative summary of inter-subject spectral variability.

The quantitative descriptors reported in Table 2 and Table 3 summarize intra-individual spectral variability across muscle–posture conditions and distance metrics under maximum power and total energy normalization. Across all metrics, NT generally exhibits higher median spectral distances than SP, reflecting greater variability, with the effect most pronounced in BR, which also shows longer IQRs compared with BB, indicating broader dispersion. Maximum power normalization amplifies these differences, resulting in higher means, STD, and CV, whereas total energy normalization produces more compact distributions and reduced dispersion.

Paired Wilcoxon signed-rank tests were used to compare SP and NT, given the non-normal distribution of spectral distances. Under maximum power normalization, posture-dependent differences reached statistical significance for several muscle–distance combinations: $L^{2}$ distances were significant for both BB (p=0.0296) and BR (p=0.0168); $L^{1}$ distances were significant for BR (p=0.0197) only; and $L^{\infty }$ distances were significant for BR (p=0.0073), with BB showing a marginal trend (p=0.0573). In contrast, total energy normalization yielded no significant differences for any muscle or distance metric (p≥0.0778), indicating that this approach stabilizes intra-subject distances and mitigates posture-related effects.

To further characterize the statistical behavior of inter-subject spectral variability, the empirical distributions of pairwise distances were examined using histograms. For each muscle–task category, all pairwise distances between participants were aggregated to form a single distribution, which reflects how frequently different levels of between-subject dissimilarity occur within the cohort.

**Figure 11:**
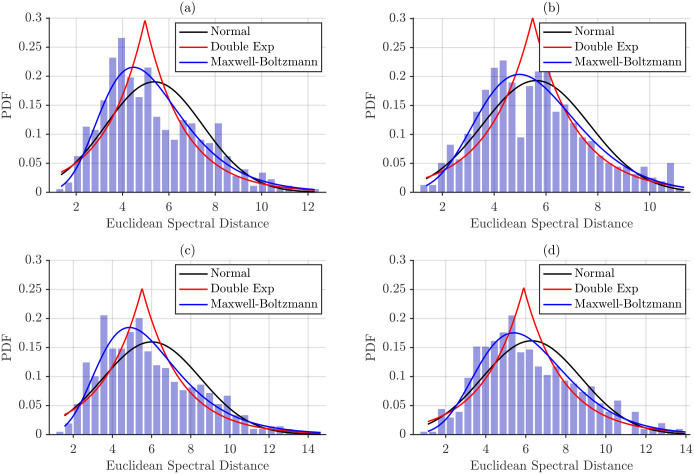
Histograms of pairwise inter-subject Euclidean distances for each muscle–task category under maximum power normalization, along with fitted probability distributions. (**a**) BB/SP, (**b**) BB/NT, (**c**) BR/SP and (**d**) BR/NT.

Figure 11 shows these histograms for spectral distances obtained using maximum power normalization. The empirical distributions were used to fit several probabilistic models—Gaussian, double exponential, and Maxwell–Boltzmann—in order to identify which model best captures the shape and spread of the observed distances.

**Figure 12:**
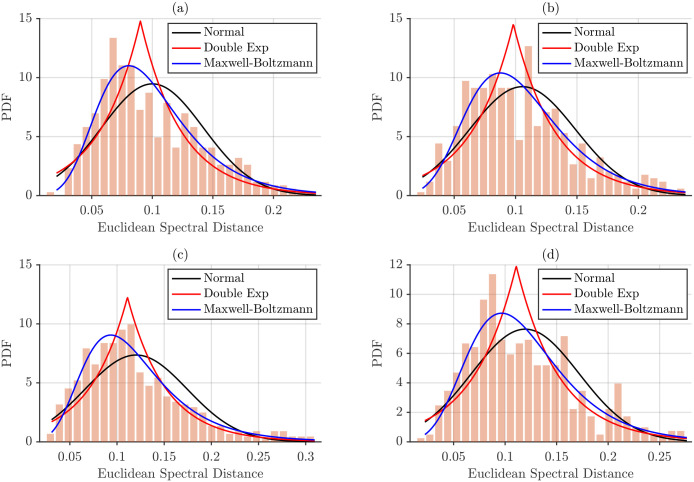
Histograms of pairwise inter-subject Euclidean distances for each muscle–task category under total energy normalization, with fitted probabilistic models. (**a**) BB/SP, (**b**) BB/NT, (**c**) BR/SP and (**d**) BR/NT.

The same procedure was applied to spectra normalized by total energy. Figure 12 presents the corresponding histograms and fitted models.

Fitted probability distribution curves, shown in Figure 11 and Figure 12, provide a visual assessment of how well each model captures the empirical inter-subject spectral distances. Across all conditions, the normal distribution consistently fails to reproduce the observed data, whereas the Maxwell–Boltzmann distribution shows the closest overall fit. Beyond differences in PDF amplitude, energy-normalized distances appear to conform more closely to the Maxwell–Boltzmann model than maximum power-normalized distances, suggesting that total energy normalization stabilizes the distribution and enhances its agreement with theoretical expectations.

The goodness-of-fit for each probability distribution was assessed using the chi-square ($\chi^{2}$) test. Results for maximum power normalization are reported in Table 6, while the corresponding results for total energy normalization are presented in Table 7. Distributions yielding p≥0.05 were considered statistically consistent with the empirical data.

The chi-squared goodness-of-fit analysis presented in Table 6 and Table 7 shows that the Normal and Double Exponential distributions are consistently rejected for all muscles, postures, and distance metrics, indicating that they do not capture the skewness and tail characteristics of the empirical inter-subject spectral distances. Under maximum power normalization, the Maxwell–Boltzmann distribution provided the most consistent fits. Acceptable fits were observed for $L^{2}$ distances in NT for both BB and BR, for $L^{1}$ distances in BB/NT and BR/SP, and for all $L^{\infty }$ distances, highlighting that inter-subject distances exhibit distributional properties such as positive support and skewness that are well described by this model.

Total energy normalization reduced the overall magnitude of inter-subject distances but produced more variable goodness-of-fit results. The Maxwell–Boltzmann distribution remained the best-fitting model for selected conditions, including BB/NT and BR/SP for $L^{2}$ distances, BB/NT and BR/SP for $L^{1}$ distances, and both postures of the BR for $L^{\infty }$ distances, while other combinations did not meet the significance threshold.

## 4. Discussion

The present study examined how forearm posture modulates the spectral characteristics of sEMG signals by analyzing posture-dependent variability at both the intra-individual and inter-individual levels. Within individuals, the results showed a systematic effect of posture, with the NT generally associated with greater spectral dispersion than the SP. This intra-individual effect was most consistent in the BB and more variable in the BR, and its magnitude depended on the chosen distance metric and normalization strategy. At the inter-individual level, spectral distances consistently deviated from Gaussian assumptions and were often compatible with Maxwell–Boltzmann distributions, suggesting that between-subject variability arises from the combined contribution of multiple spectral components rather than from a single stochastic process.

These findings align closely with the current understanding of neuromuscular coordination. A substantial body of literature has shown that EMG signals from synergistic muscles exhibit coherent oscillatory activity, reflecting shared synaptic input to motor neuron pools. Coherence analysis during rhythmic and force-controlled tasks has demonstrated that such common neural drive adapts to task demands and biomechanical context, shaping both coordination patterns and the spectral characteristics of EMG signals [32,33]. More recently, it has been shown that the spectral composition of synaptic input to motor neurons is explicitly task-dependent, adjusting to functional requirements rather than remaining fixed across conditions [34]. Within this framework, the posture-related spectral differences observed here are consistent with context-dependent modulation of neural drive and its transformation into surface EMG.

The robust NT > SP effect observed in the BB across all distance metrics ($L^{2}$, $L^{1}$, and $L^{\infty }$) and both normalization schemes indicates a strong sensitivity of this muscle to forearm posture. Changes in joint configuration alter mechanical leverage, tendon compliance, and afferent feedback, all of which can influence how common synaptic input is distributed across motor units and how this activity is expressed in the surface EMG spectrum. Moreover, studies of motor-unit behavior show that recruitment strategies and shared synaptic input adapt to mechanical and functional demands, and that joint configuration can modulate both the degree of common drive and its transmission to the surface EMG signal [35]. In the BB, recruitment patterns are also strongly influenced by contraction level, which may interact with posture to amplify spectral differences [36]. Taken together, these physiological mechanisms help explain why the BB shows a robust and consistent posture effect, reflecting a modulation of the statistics of neural drive and its biomechanical expression rather than a deterministic control strategy.

In contrast, posture-related effects in the BR were less uniform. Although NT generally produced higher spectral variability than SP, certain combinations of distance metrics and normalization strategies showed weaker or nonsignificant differences. Notably, under the $L^{\infty }$ metric with total energy normalization, the typical NT > SP trend was reversed, with SP exhibiting slightly higher spectral variability. This isolated inversion likely reflects the sensitivity of the $L^{\infty }$ metric to localized spectral peaks, which can occasionally dominate the measure and mask distributed broadband changes. In comparison, $L^{1}$ and $L^{2}$, which integrate deviations across the full frequency range, were more sensitive to posture-related effects. This observation mirrors expectations from coherence and motor-unit studies, where task- or posture-induced changes in neural drive tend to be distributed across frequency bands rather than confined to isolated peaks.

Importantly, posture should not be interpreted as a unilateral cause of activation changes. The relationship between posture and muscle activation is inherently bidirectional: joint configuration influences neural control through biomechanics and sensory feedback, while muscle activation simultaneously stabilizes and constrains posture. The present study quantified spectral differences between two fixed postures but did not isolate causal mechanisms. Establishing causality would require experimental designs that decouple posture from neural drive, such as matched-torque tasks across joint angles or motor-unit decomposition under externally stabilized conditions.

Participant demographic characteristics such as age, sex, height, body mass, and BMI were recorded (Table 1). While this study did not formally assess their influence on EMG outcomes, the sample was relatively homogeneous and representative of young adults. Reporting these data provides context for the participants studied and may guide future work examining potential links between demographics and neuromuscular activation patterns.

A notable methodological outcome concerns the statistical structure of inter-subject variability. The systematic rejection of Gaussian and double-exponential models indicates that EMG spectral distances do not follow standard symmetric or simple heavy-tailed distributions. Their frequent compatibility with Maxwell–Boltzmann models suggests that inter-subject variability behaves like the magnitude of a multivariate deviation, consistent with variability arising from multiple independent spectral contributions.

Several factors likely contributed to the failure of standard distributions to fit the empirical histograms of spectral distances. Surface EMG signals are well known to exhibit non-Gaussian behavior influenced by physiological conditions and contraction levels. Empirical research has shown that the probability density function of sEMG may be super-Gaussian, Laplacian, or may evolve toward Gaussianity only at higher contraction forces, with shape oscillations at low force levels due to individual-specific neuromuscular features. These complex changes across force conditions highlight why simple symmetric models cannot capture the true distributional structure of sEMG. Moreover, single-Gaussian models tend to be inadequate because sEMG recordings frequently reflect mixtures of underlying random processes with variable variance. In fact, modern statistical approaches based on scale-mixture or compound models have been proposed precisely because they better represent the non-Gaussian nature of sEMG and the variability of variance linked to motor unit behavior [37].

In addition, sEMG is inherently non-stationary and nonlinear, even during isometric efforts. Its properties evolve due to motor unit recruitment, firing variability, fatigue-related changes, and other physiological factors. These variations alter spectral content over time and violate the assumptions of stationarity required by many parametric models [38]. Nonlinear analyzes have repeatedly shown that sEMG contains deterministic nonlinear components and exhibits dynamical complexity, including chaotic and multifractal traits under some conditions. These physiologically driven non-stationarities and nonlinearities contribute to multimodal spectral distance distributions that are not well described by simple symmetric or heavy-tailed forms. Such evidence supports the use of non-parametric statistical methods and motivates future probabilistic modeling based on Maxwell–Boltzmann or mixture-based representations [39].

From an applied perspective, the results highlight key methodological considerations. Maximum power normalization enhanced sensitivity to posture-related differences, particularly in the BR, likely by emphasizing amplitude-dominant spectral components that are sensitive to biomechanical configuration. Total energy normalization produced more conservative outcomes and may be preferable when reducing posture sensitivity is desired. Among distance metrics, $L^{1}$ and $L^{2}$ provided the most reliable discrimination of posture effects, whereas $L^{\infty }$ primarily reflected localized spectral irregularities. The substantial inter-subject coefficients of variation further emphasize the importance of adequate sample sizes and robust statistical summaries in EMG studies.

## 5. Limitations of the Study

This study provides novel insights into posture-related spectral variability in sEMG signals, but several methodological and interpretative limitations should be considered. The participant sample consisted of 33 young, healthy, and demographically homogeneous individuals. While comparable to similar studies, this limits the generalizability of the findings, as spectral patterns may differ in older adults, clinical populations, or individuals with impaired neuromuscular control. Future work should therefore include larger and more diverse cohorts to capture a broader spectrum of neuromuscular behavior.

The tasks were performed at near-maximal voluntary contraction without direct force measurement or real-time feedback. Because sEMG spectral features are influenced by contraction intensity and force output may vary with posture and individual activation strategies, the lack of controlled or quantified force may contribute to variability in the observed spectral measures. Forearm posture can affect muscle length, moment arm, and activation patterns, which also contribute to force generation and joint stability. Therefore, the present design allows for the description of posture-related spectral differences but does not fully separate the effects of posture and force. Future studies that control or match force levels across postures could help clarify these effects.

The followed approach for signal segmentation relied on manually tuned parameters and deterministic rules set for controlled isometric tasks. Although effective within the present dataset, this approach may have limited robustness across different populations, tasks, or noise conditions. Alternative strategies, especially advanced machine-learning–based activation detection [40], could improve generalizability.

Repeated contractions per condition may have introduced localized fatigue, which was not explicitly monitored. Even short-duration isometric efforts can subtly alter spectral content, so some observed variability may reflect fatigue rather than posture alone. Incorporating fatigue monitoring or contraction-level fatigue indices in future studies would help isolate posture-specific effects more clearly.

Finally, although normalized spectral representations effectively distinguished postures and conditions in this controlled setting, these results may not generalize to tasks involving substantial fatigue, dynamic movements, or non-isometric contractions. Future work could also benefit from comparing alternative analytical frameworks, such as coherence- or motor-unit–based metrics, to deepen the understanding of neuromuscular variability. The findings should be interpreted as an initial demonstration of posture-related spectral modulation rather than universally applicable descriptors of muscle function.

## 6. Conclusions

The present study aimed to characterize how forearm posture influences the spectral variability of surface EMG signals in elbow flexor muscles, with a broader focus on intra- and inter-individual consistency in neuromuscular activation. sEMG recordings from the biceps brachii and brachioradialis of 33 healthy adults were collected during repeated near-maximal isometric elbow flexion tasks performed with supinated and neutral forearm orientations. Power spectra were smoothed using a moving-trend filter and normalized using both maximum and total-energy criteria, allowing posture-dependent differences to be quantified using Minkowski distance metrics at the individual and group levels.

The results suggested that forearm posture may influence EMG spectral patterns. The biceps brachii generally exhibited greater spectral variability in the neutral posture across all distance metrics, normalization schemes, and levels of variability. This pattern was observed in both intra- and inter-subject comparisons and was most pronounced when using Manhattan and Euclidean distances with maximum-based normalization. The brachioradialis also showed posture-dependent variability, although the effect was less consistent and more sensitive to analytical choices. These observations indicate that spectral variability differs between muscles and likely reflects the combined influence of neural and biomechanical factors associated with forearm orientation, while also potentially being affected by variations in contraction intensity, fatigue, or task-specific biomechanics.

Analysis of the statistical structure of inter-subject distances revealed additional insights. Models based on Gaussian and Laplace distributions were consistently inadequate, whereas several combinations of posture, muscle, and normalization were reasonably captured by Maxwell–Boltzmann distributions. This pattern suggests that inter-subject spectral differences may behave like multivariate resultant magnitudes, supporting the application of nonparametric inference and motivating the future development of generative models based on Maxwell–Boltzmann distributions to describe neuromuscular variability.

Although this study provides novel insights into posture-related spectral variability in elbow flexor muscles, the findings are based on a relatively homogeneous sample of young, healthy adults performing near-maximal isometric contractions without direct force monitoring. Therefore, the observed patterns should be interpreted as reflecting posture-associated trends that may also be influenced by force output variability, fatigue, and task-specific biomechanical factors, rather than solely representing causal effects of forearm orientation.

## Figures and Tables

**Table 1:** Anthropometric characteristics of the study participants (mean ± SD).

Parameter	Total (n = 33)	Females (n = 18)	Males (n = 15)
Age [years]	23.0 ± 0.6	24.0 ± 0.7	22.0 ± 0.9
Height [cm]	173.8 ± 8.4	167.5 ± 5.8	180 ± 6.2
Body mass [kg]	71.5 ± 9.5	63.5 ± 6.2	80.0 ± 8.1
BMI [kg/m^2^]	23.5 ± 2.8	22.6 ± 2.4	24.4 ± 3.1

**Table 2:** Statistics of intra-subject distances (Maximum power normalization).

Distance	Muscle	Posture	Mean	STD	CV	Min	Max
$L^{2}$	Biceps brachii	SP	3.2024	0.51481	0.16076	2.1621	4.2828
		NT	3.4212	0.46235	0.13514	2.4942	4.1698
	Brachioradialis	SP	3.2563	0.65395	0.20083	2.1574	4.3949
		NT	3.5612	0.66527	0.18681	2.3604	4.7256
$L^{1}$	Biceps brachii	SP	88.629	16.78	0.18933	53.955	118.45
		NT	94.955	17.287	0.18205	62.688	127.64
	Brachioradialis	SP	91.303	25.039	0.27424	53.662	141.57
		NT	100.6	29.048	0.28875	62.073	160.28
$L^{\infty }$	Biceps brachii	SP	0.23305	0.031371	0.13461	0.16445	0.29082
		NT	0.24345	0.032027	0.13155	0.18479	0.31318
	Brachioradialis	SP	0.23354	0.038643	0.16547	0.16461	0.31578
		NT	0.2521	0.035815	0.14206	0.16766	0.31352

Wilcoxon signed-rank test (*p*-values): $L^{2}$: Biceps brachii (p=0.0296), Brachioradialis (p=0.0168), $L^{1}$: Biceps brachii (p=0.0997), Brachioradialis (p=0.0197), $L^{\infty }$: Biceps brachii (p=0.0573), Brachioradialis (p=0.0073).

**Table 3:** Statistics of intra-subject distances (Total energy normalization).

Distance	Muscle	Posture	Mean	STD	CV	Min	Max
$L^{2}$	Biceps brachii	SP	0.055649	0.007407	0.1331	0.041384	0.068177
		NT	0.058691	0.0097327	0.16583	0.043595	0.08532
	Brachioradialis	SP	0.058899	0.011728	0.19912	0.042329	0.097856
		NT	0.059482	0.0093096	0.15651	0.040107	0.078016
$L^{1}$	Biceps brachii	SP	1.4843	0.18718	0.12611	1.0873	1.8455
		NT	1.5653	0.21971	0.14037	1.1658	2.1452
	Brachioradialis	SP	1.5664	0.2593	0.16554	1.165	2.2791
		NT	1.5948	0.22075	0.13842	1.1207	1.9713
$L^{\infty }$	Biceps brachii	SP	0.0043532	0.00064703	0.14863	0.0030485	0.0056983
		NT	0.0044657	0.00095119	0.213	0.0031854	0.0073217
	Brachioradialis	SP	0.0046096	0.0011857	0.25722	0.0029947	0.0089992
		NT	0.0045563	0.00079049	0.1735	0.0031827	0.006094

Wilcoxon signed-rank test (*p*-values): $L^{2}$: Biceps brachii (p=0.0778), Brachioradialis (p=0.4805), $L^{1}$: Biceps brachii (p=0.1038), Brachioradialis (p=0.4805), $L^{\infty }$: Biceps brachii (p=0.5435), Brachioradialis (p=0.7539).

**Table 4:** Statistics of inter-subject distances (Maximum power normalization).

Distance	Muscle	Posture	Mean	STD	CV	Min	Max
$L^{2}$	Biceps brachii	SP	0.23305	0.031371	0.13461	0.16445	0.29082
		NT	0.24345	0.032027	0.13155	0.18479	0.31318
	Brachioradialis	SP	0.23354	0.038643	0.16547	0.16461	0.31578
		NT	0.2521	0.035815	0.14206	0.16766	0.31352
$L^{1}$	Biceps brachii	SP	156.96	60.216	0.38365	51.834	368.66
		NT	173.63	66.935	0.38551	52.864	408.72
	Brachioradialis	SP	192.24	91.593	0.47645	38.547	528.58
		NT	204.23	96.08	0.47045	40.235	519.56
$L^{\infty }$	Biceps brachii	SP	0.33401	0.15142	0.45335	0.073086	0.88544
		NT	0.33598	0.13225	0.39364	0.068593	0.73016
	Brachioradialis	SP	0.32661	0.12332	0.37758	0.10087	0.73247
		NT	0.33346	0.12035	0.36092	0.065016	0.72578

Wilcoxon signed-rank test (*p*-values): $L^{2}$: Biceps brachii (p=0.0019), Brachioradialis (p=0.0346), $L^{1}$: Biceps brachii (p=0.0000), Brachioradialis (p=0.0014), $L^{\infty }$: Biceps brachii (p=0.1707), Brachioradialis (p=0.3360).

**Table 5:** Statistics of inter-subject distances (Total energy normalization).

Distance	Muscle	Posture	Mean	STD	CV	Min	Max
$L^{2}$	Biceps brachii	SP	0.0043532	0.00064703	0.14863	0.0030485	0.0056983
		NT	0.0044657	0.00095119	0.213	0.0031854	0.0073217
	Brachioradialis	SP	0.0046096	0.0011857	0.25722	0.0029947	0.0089992
		NT	0.0045563	0.00079049	0.1735	0.0031827	0.006094
$L^{1}$	Biceps brachii	SP	2.8595	1.0922	0.38194	0.86022	6.0835
		NT	3.1318	1.2254	0.39129	0.8392	7.0932
	Brachioradialis	SP	3.5488	1.4888	0.41953	0.85423	8.5023
		NT	3.7088	1.6374	0.4415	0.79132	8.485
$L^{\infty }$	Biceps brachii	SP	0.0073168	0.0037904	0.51804	0.0012855	0.019271
		NT	0.0072639	0.0033466	0.46072	0.0018256	0.017325
	Brachioradialis	SP	0.0083701	0.0043484	0.51952	0.0019441	0.024539
		NT	0.00761	0.0034491	0.45323	0.0014163	0.019085

Wilcoxon signed-rank test (*p*-values): $L^{2}$: Biceps brachii (p=0.5435), Brachioradialis (p=0.7539), $L^{1}$: Biceps brachii (p=0.0001), Brachioradialis (p=0.0170), $L^{\infty }$: Biceps brachii (p=0.5407), Brachioradialis (p=0.0000).

**Table 6:** Chi-squared goodness-of-fit test results for inter-subject distances under maximum power normalization. Bold indicates p≥0.05.

Distance	Muscle	Posture	Normal	Double Exp	Maxwell–Boltzmann
$L^{2}$	Biceps brachii	SP	0.0000	0.0000	0.0093
		NT	0.0003	0.0000	**0.8959**
	Brachioradialis	SP	0.0000	0.0000	0.0001
		NT	0.0000	0.0000	**0.0659**
$L^{1}$	Biceps brachii	SP	0.0000	0.0000	0.0057
		NT	0.0002	0.0000	**0.5110**
	Brachioradialis	SP	0.0000	0.0000	**0.8879**
		NT	0.0000	0.0000	0.7081
$L^{\infty }$	Biceps brachii	SP	0.0000	0.0000	**0.4449**
		NT	0.0000	0.0000	**0.8011**
	Brachioradialis	SP	0.0000	0.0000	**0.2032**
		NT	0.0002	0.0000	**0.6075**

**Table 7:** Chi-squared goodness-of-fit test results for inter-subject distances under total energy normalization. Bold indicates p≥0.05.

Distance	Muscle	Condition	Normal	Double Exp	Maxwell–Boltzmann
$L^{2}$	Biceps brachii	SP	0.0000	0.0000	0.0040
		NT	0.0000	0.0000	**0.2063**
	Brachioradialis	SP	0.0000	0.0000	**0.4869**
		NT	0.0000	0.0000	0.0471
$L^{1}$	Biceps brachii	SP	0.0000	0.0000	0.0195
		NT	0.0000	0.0000	**0.7454**
	Brachioradialis	SP	0.0000	0.0000	**0.9707**
		NT	0.0000	0.0000	0.0035
$L^{\infty }$	Biceps brachii	SP	0.0000	0.0000	0.0062
		NT	0.0000	0.0000	0.0014
	Brachioradialis	SP	0.0000	0.0000	**0.7175**
		NT	0.0000	0.0000	**0.3696**

## Data Availability

The original data presented in the study, as well as the full analysis pipeline, are openly available at the repository: https://github.com/informacja/Collegium-Medicum/tree/main/EMG-Study (accessed on 25 January 2026).

## Supplementary Materials

The following supporting information can be downloaded at: https://www.mdpi.com/article/10.3390/s26030878/s1.
