# Supplementary material for: Intra- and Inter-Individual Spectral Pattern Variability of sEMG in Elbow Flexor Motor Tasks
Source: Sensors (Basel). 2026 Jan 29;26(3):878. doi: 10.3390/s26030878 (PMC12899053; doi:10.3390/s26030878)
Supplement: Supplementary file 1 [file sensors-26-00878-s001.zip › sensors-4076709-supplementary.pdf]

L1 – Manhattan Intra

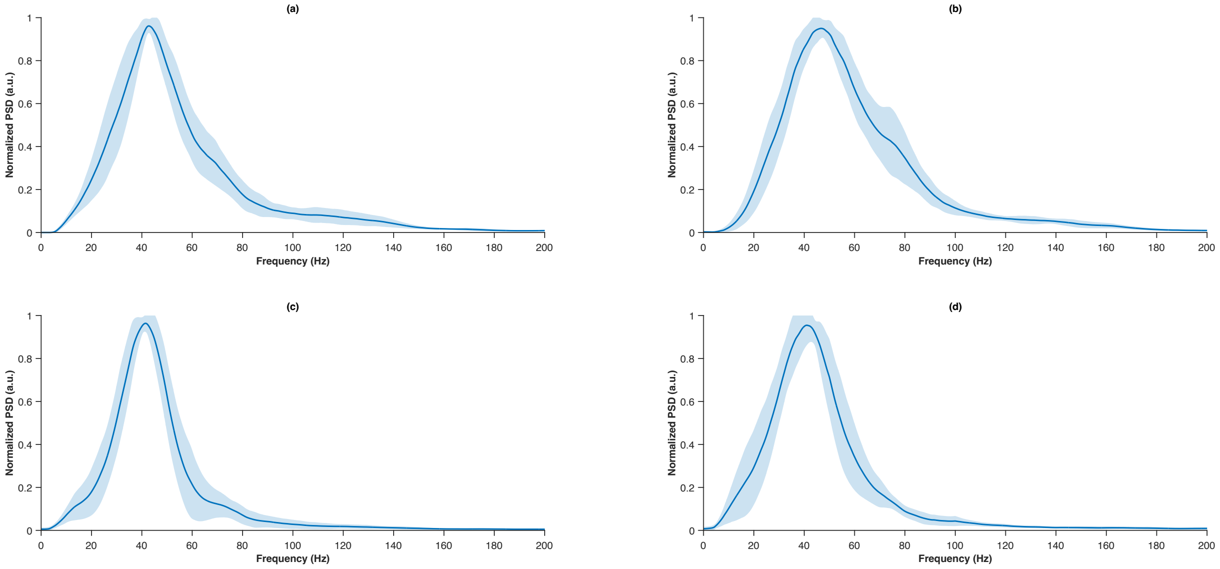

Figure S 1. Average power spectra of a representative participant under maximum-power normalization for all muscle–task conditions: (a) BB/SP, (b) BB/NT, (c) BR/SP, and (d) BR/NT. Shaded area represents the mean ± STD.

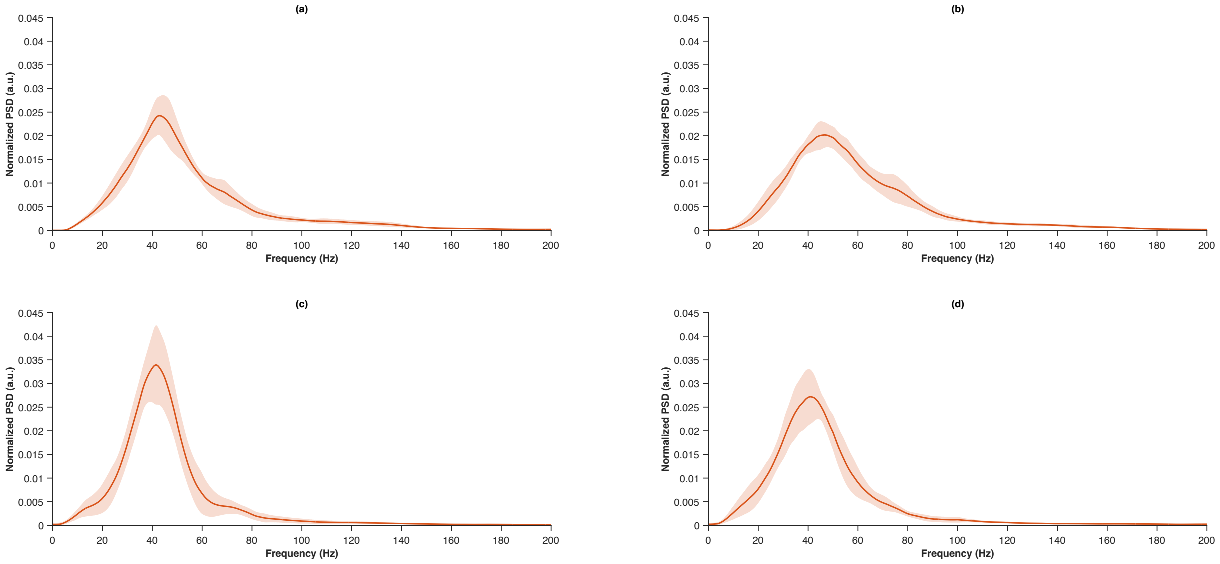

Figure S2. Average power spectra of a representative participant under energy normalization for all muscle–task conditions: (a) BB/SP, (b) BB/NT, (c) BR/SP, and (d) BR/NT. Shaded area represents the mean ± STD.

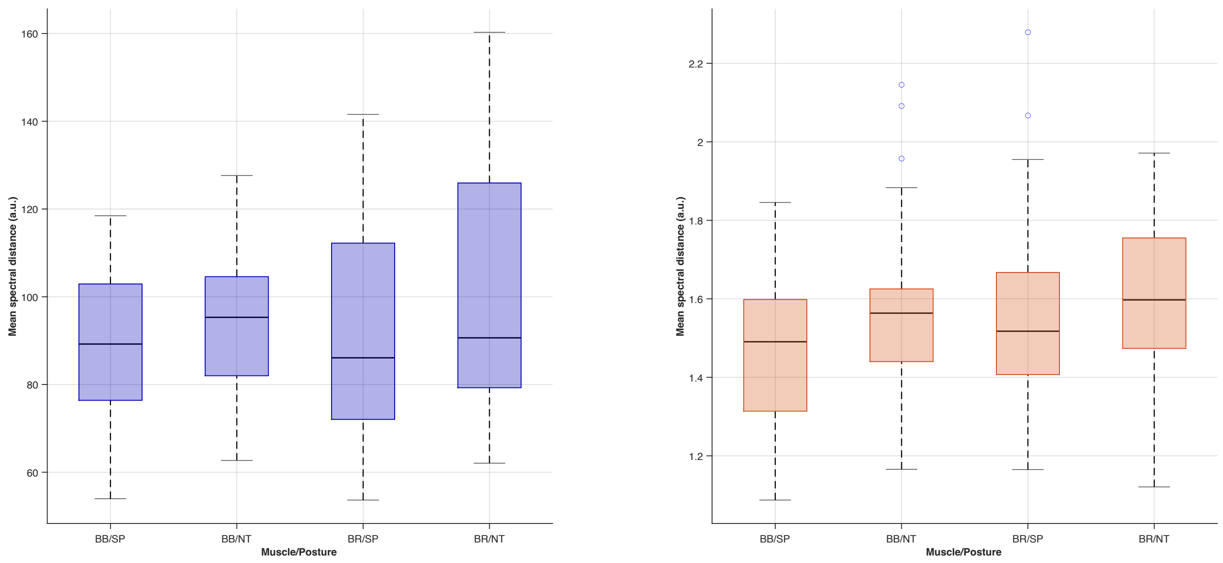

Figure S3. Boxplots showing intra-individual spectral variability across muscle–task categories. (a) Maximum-power normalization and (b) Energy normalization.

Manchatan L1 – INTER

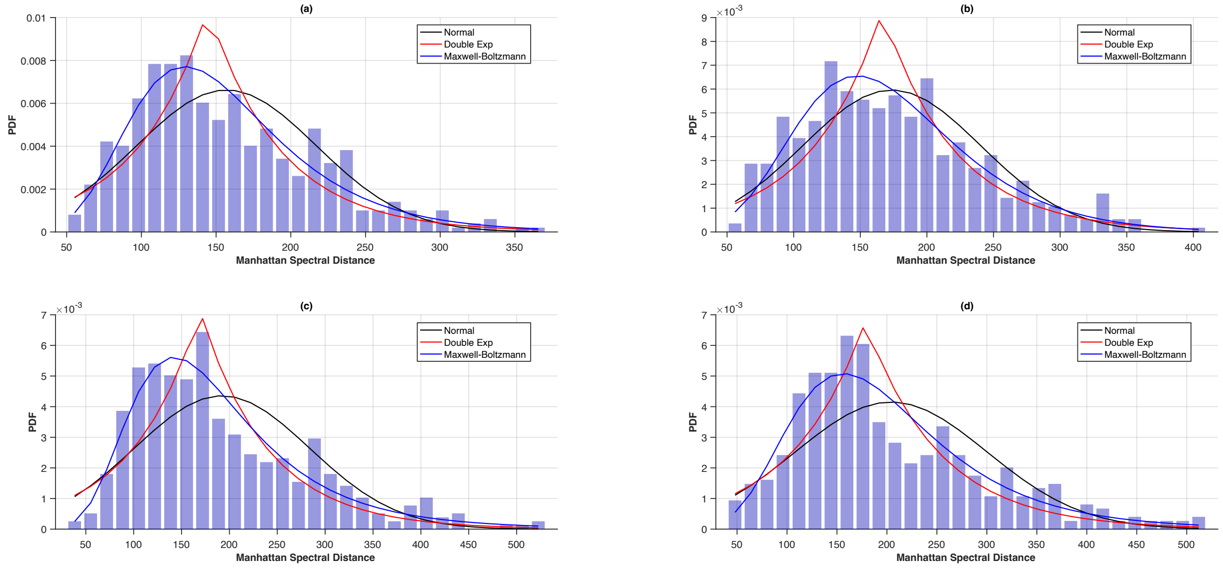

Figure S4. Histograms of pairwise inter-subject Euclidean distances for each muscle–task category under maximum-power normalization, along with fitted probability distributions. (a) BB/SP, (b) BB/NT, (c) BR/SP and (d) BR/NT.

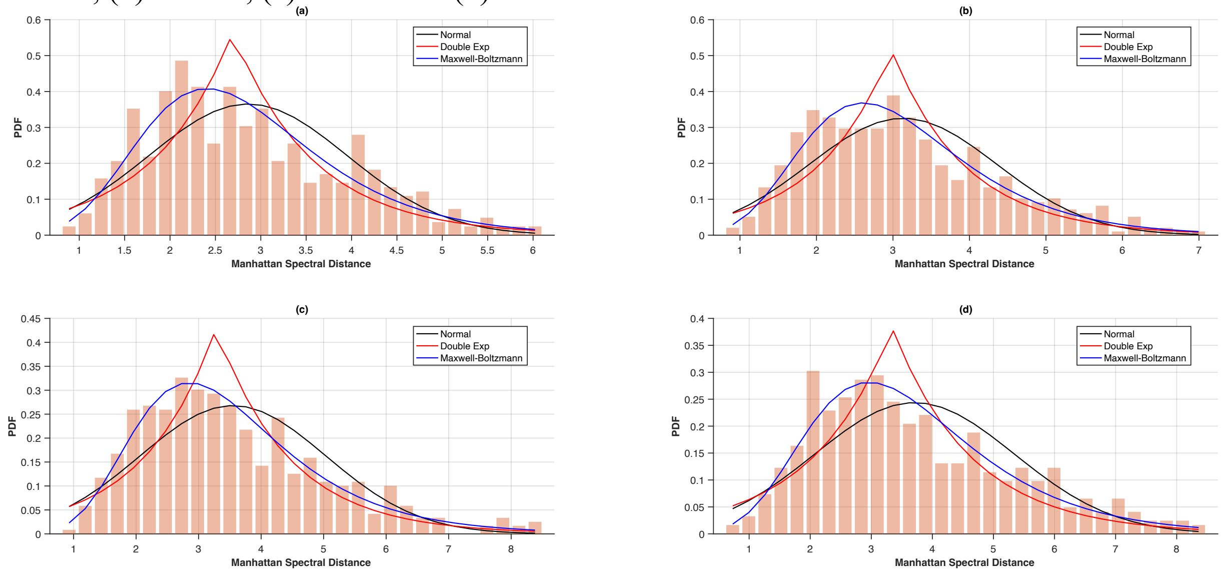

Figure S 5. Histograms of pairwise inter-subject Euclidean distances for each muscle–task category under energy normalization, with fitted probabilistic models. (a) BB/SP, (b) BB/NT, (c) BR/SP and (d) BR/NT.

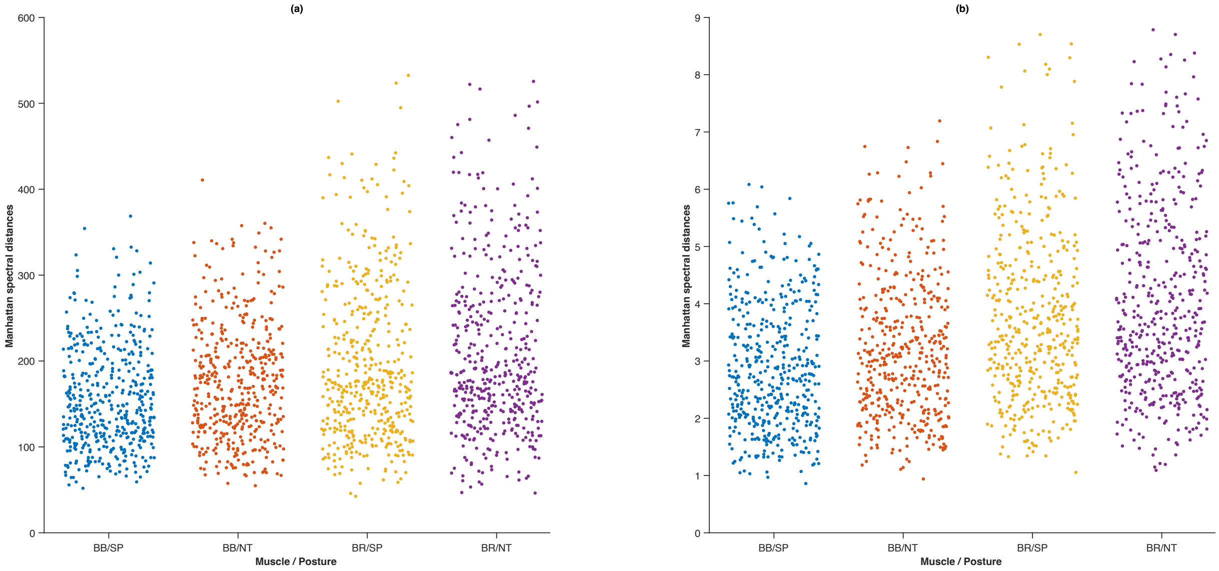

Figure S6. Pairwise inter-subject spectral distances for all muscle–task categories: (a) maximum-power normalization and (b) energy normalization. Vertical offsets are applied only for visual separation of categories.

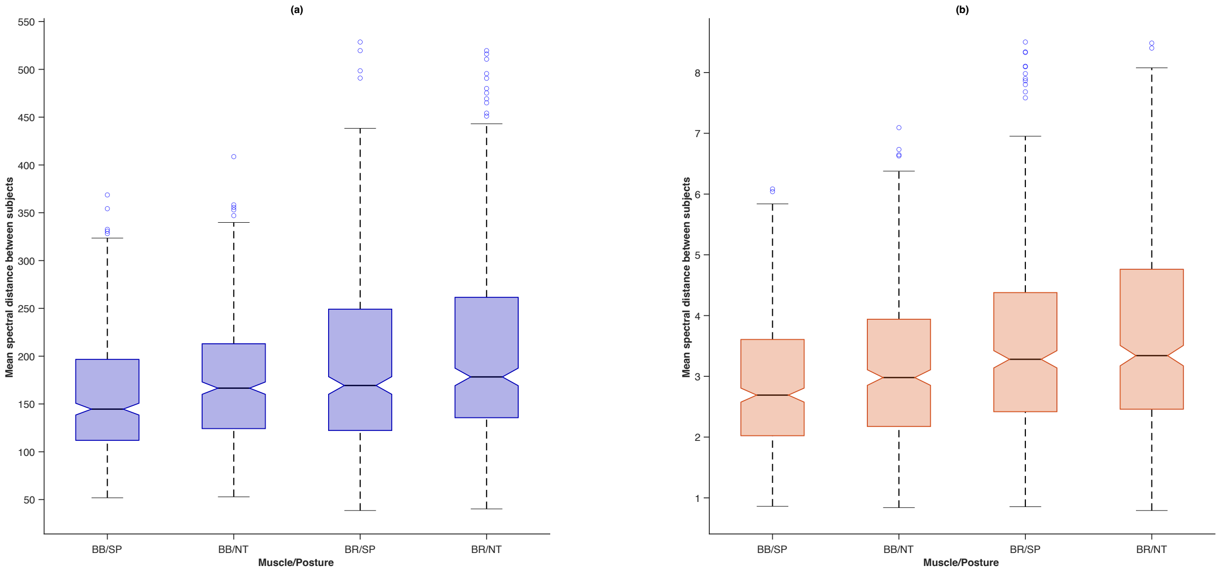

Figure S7. Boxplots showing inter-individual spectral variability across muscle–task categories. (a) maximum-power normalization and (b) energy normalization.

L3 Chebyshew Intra

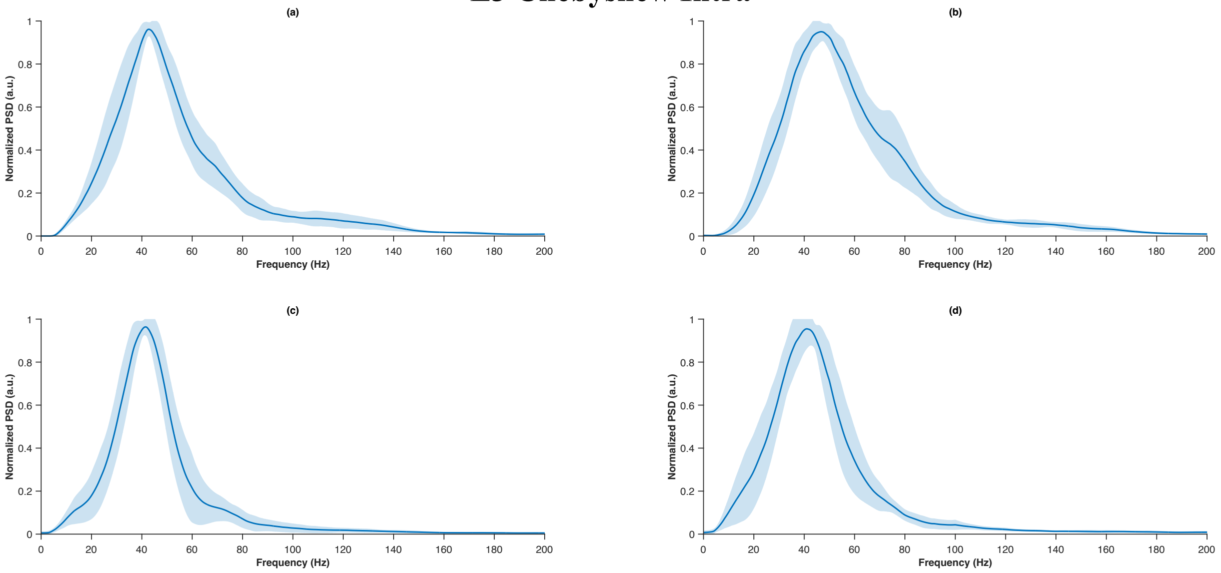

Figure S 8. Average power spectra of a representative participant under maximum-power normalization for all muscle–task conditions: (a) BB/SP, (b) BB/NT, (c) BR/SP, and (d) BR/NT. Shaded area represents the mean ± STD.

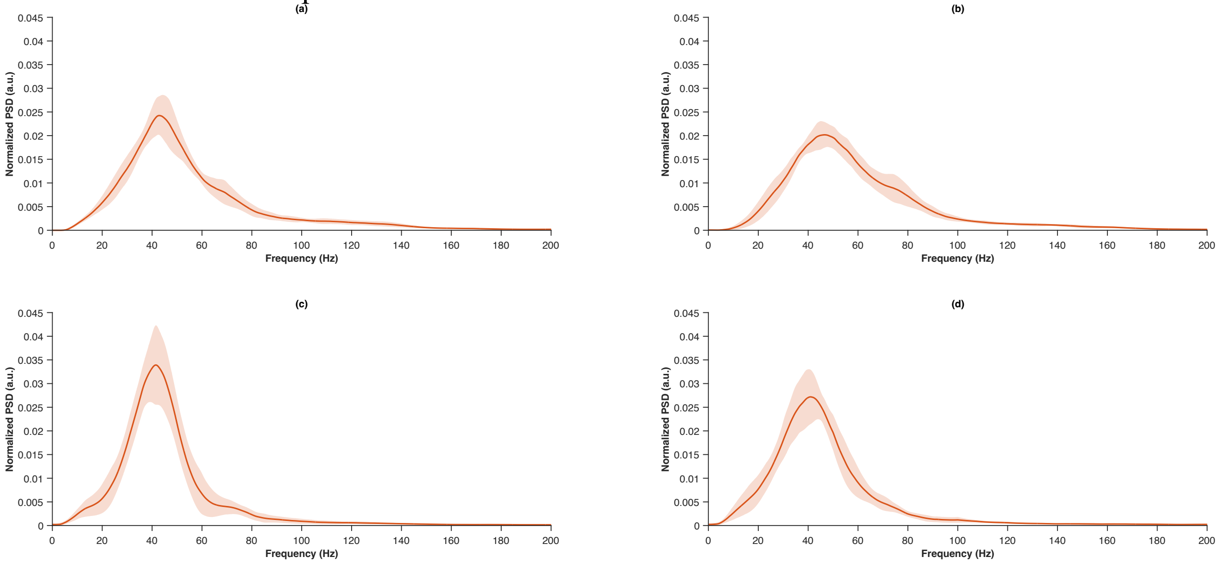

Figure S9. Average power spectra of a representative participant under energy normalization for all muscle–task conditions: (a) BB/SP, (b) BB/NT, (c) BR/SP, and (d) BR/NT. Shaded area represents the mean ± STD.

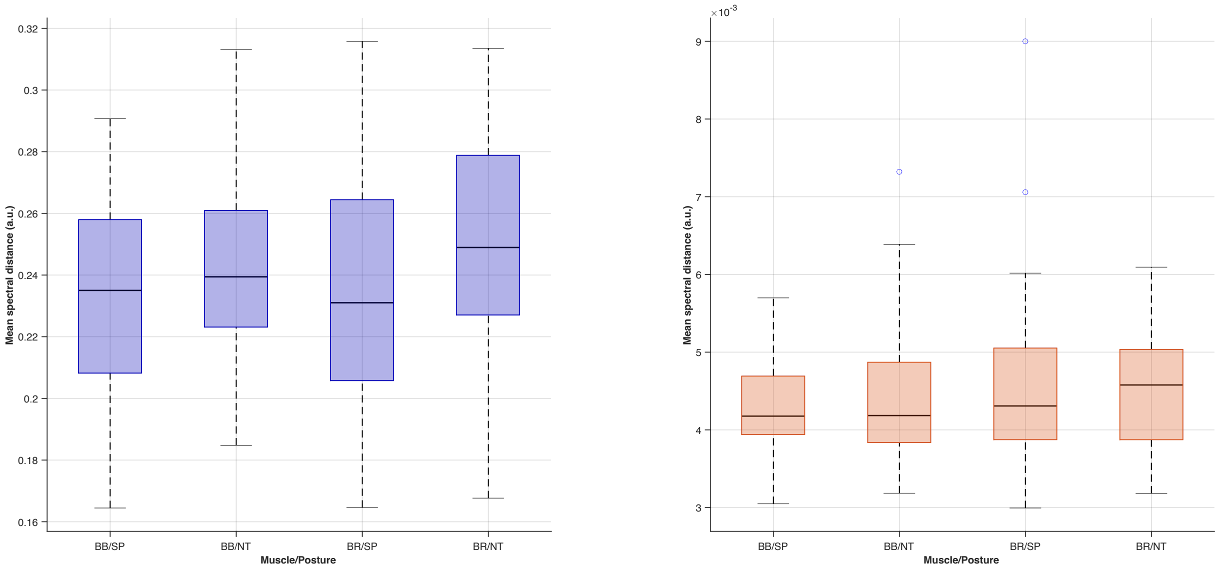

Figure S10. Boxplots showing intra-individual spectral variability across muscle–task categories. (a) Maximum-power normalization and (b) Energy normalization.

INTER – Chebyshew L3

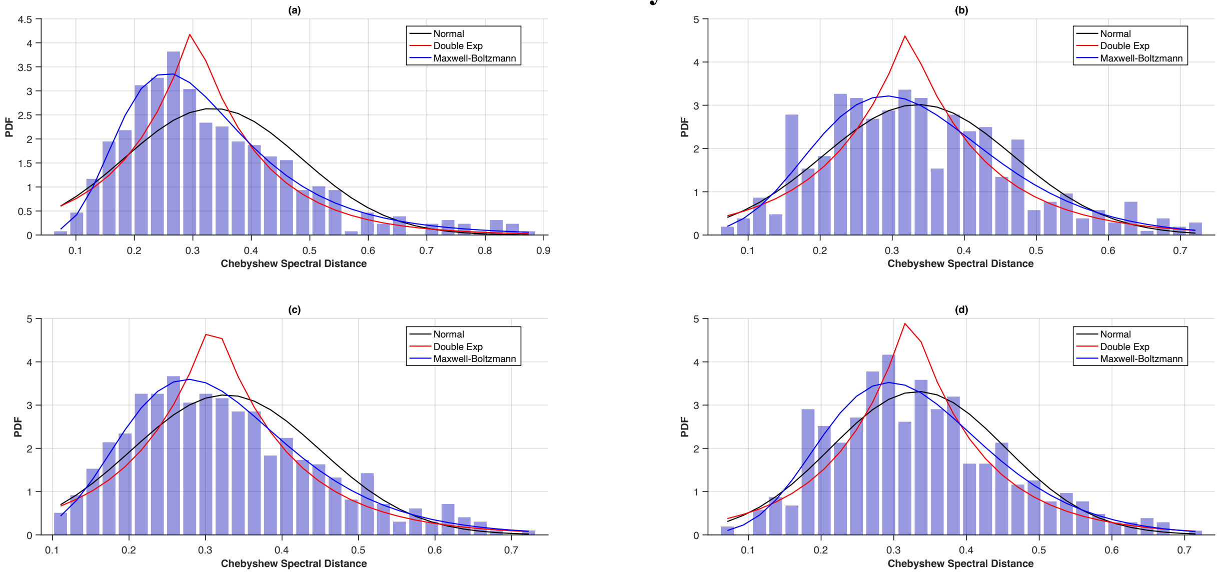

Figure S11. Histograms of pairwise inter-subject Euclidean distances for each muscle–task category under maximum-power normalization, along with fitted probability distributions. (a) BB/SP, (b) BB/NT, (c) BR/SP and (d) BR/NT.

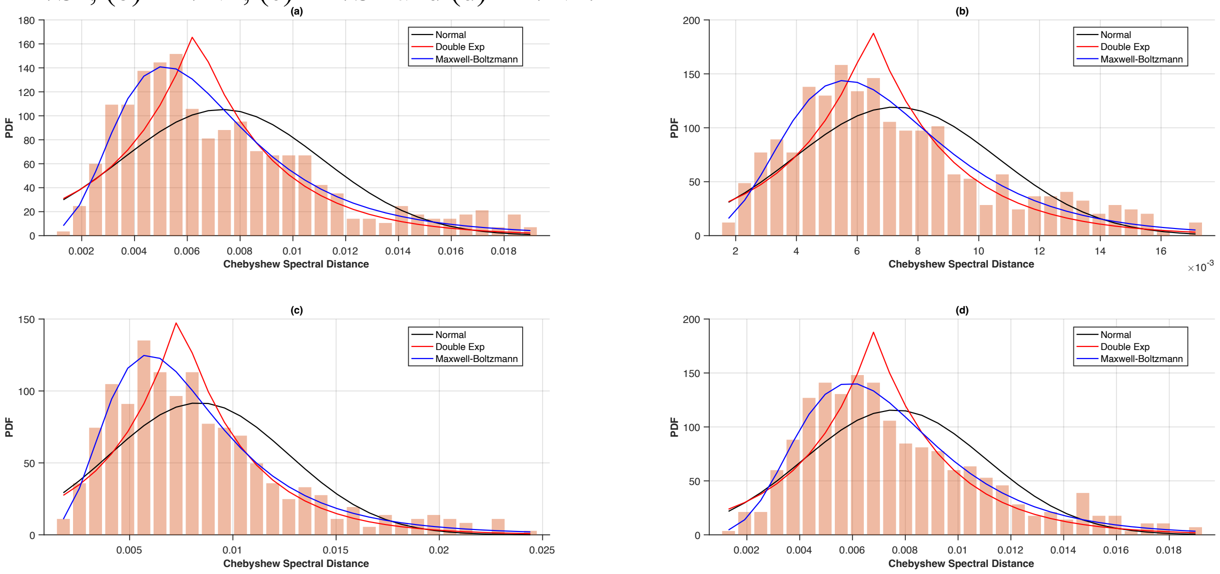

Figure S 12. Histograms of pairwise inter-subject Euclidean distances for each muscle–task category under energy normalization, with fitted probabilistic models. (a) BB/SP, (b) BB/NT, (c) BR/SP and (d) BR/NT.

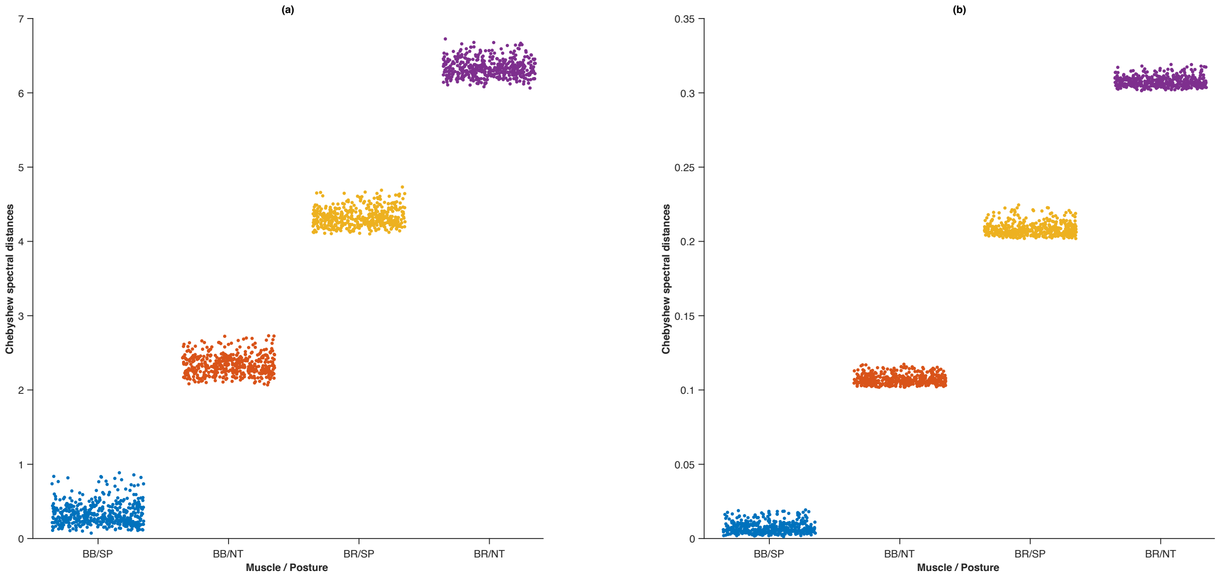

Figure S 13. Pairwise inter-subject spectral distances for all muscle–task categories: (a) maximum-power normalization and (b) energy normalization. Vertical offsets are applied only for visual separation of categories.

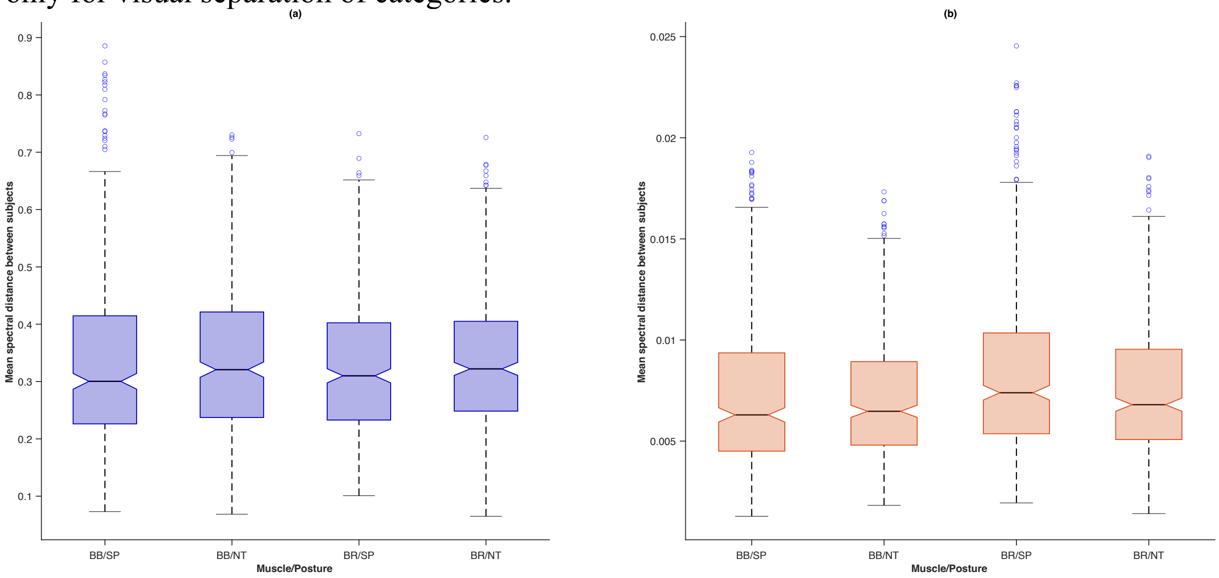

Figure S14. Boxplots showing inter-individual spectral variability across muscle–task categories. (a) maximum-power normalization and (b) energy normalization.
